# Physicochemical and Functional Characterization of a Novel Extremophilic Exopolysaccharide Produced by *Janibacter limosus*

**DOI:** 10.3390/microorganisms14091983

**Published:** 2026-09-08

**Authors:** Chaima Farhat, Patrícia Concórdio Reis, Besma Ettoumi, Kleyde Ramos, Ameur Cherif, Filomena Freitas, Habib Chouchane

**Affiliations:** 1 Laboratory of Biotechnology and Valorization of Bio-Geo Resources (BVBGR-LR11ES31), Higher Institute of Biotechnology of Sidi Thabet (ISBST), BiotechPole of Sidi Thabet, University of Manouba, Ariana 2020, Tunisia; chaymafarhat027@gmail.com (C.F.); ameur.cherif@uma.tn (A.C.); 2 UCIBIO—Applied Molecular Biosciences Unit, Department of Chemistry, School of Science and Technology, NOVA University Lisbon, 2829-516 Caparica, Portugal; pc.reis@fct.unl.pt (P.C.R.); kd.ramos@campus.fct.unl.pt (K.R.); a4406@fct.unl.pt (F.F.); 3 Associate Laboratory i4HB—Institute for Health and Bioeconomy, School of Science and Technology, NOVA University Lisbon, 2829-516 Caparica, Portugal; 4 Department of Food, Environmental and Nutritional Sciences (DeFENS), University of Milan, 20133 Milan, Italy; besma.ettoumi@gmail.com

**Keywords:** extremophilic exopolysaccharides, marine actinobacteria, *Janibacter limosus*, characterization, thermostability, rheology

## Abstract

Extremophilic exopolysaccharides (EPSs) from extreme-environment microorganisms exhibit multifunctional and specific properties relevant to food, pharmaceutical, and environmental applications. This study reports the physicochemical characteristics of a novel EPS secreted by the marine actinobacterium *Janibacter limosus* (JlEPS) isolated from the Tyrrhenian Sea. The biopolymer was characterized for its molecular mass, monosaccharide composition, functional groups, rheological, and thermal behavior. JlEPS was found as an acidic heteropolysaccharide composed of eight sugar monomers. Neutral and acidic sugars predominate, with glucose (36.60 ± 0.46 mol%), rhamnose (23.62 ± 0.15 mol%), and galacturonic acid (16.54 ± 0.18 mol%). Amino sugars were also detected, including galactosamine (8.70 ± 0.01 mol%) and glucosamine (4.00 ± 0.10 mol%). Arabinose (7.8 ± 0.76 mol%), galactose (1.35 ± 0.01 mol%), and fucose (1.42 ± 0.06 mol%) were present in minor amounts. The average molecular weight (M_w_) of the biopolymer was 1.60 × 10^6^ Da, with a polydispersity index of 1.022, indicating a relatively homogeneous population. FT-IR indicated uronic acids and glycosidic linkages, while TGA demonstrated stability up to 250 °C. JlEPS solutions behaved as pseudoplastic fluids, and the flow curves were fitted with the Carreau model (R^2^ = 0.96–0.99). The zero-shear viscosity increased from 0.542 ± 0.187 Pa·s (0.5%) to (5.01 ± 0.91) × 10^4^ Pa·s, while the flow index decreased from 0.368 ± 0.032 to 0.100 ± 0.028, confirming strong pseudoplasticity and extensive molecular entanglements. JlEPS is a complex with a defined composition and high viscosity, supporting its potential for multiple applications.

## 1. Introduction

Microorganisms such as fungi, microalgae, and bacteria secrete high-molecular-weight polysaccharides known as exopolysaccharides (EPSs) that serve various structural and ecological roles [1]. Owing to their structural diversity and versatile functional groups, EPSs have gained increasing attention for their functional properties in different sectors [2,3,4,5,6]. Structurally, EPSs are generally classified into homopolysaccharides, composed of repeating units of a single monosaccharide, and heteropolysaccharides, consisting of different monosaccharides arranged in complex sequences. Their main structural determinants, including the type of glycosidic linkage, degree of branching, molecular weight, and functional groups, play a crucial role in defining their physicochemical properties and biological functions [7]. Among these physicochemical aspects, the rheological properties of EPSs are particularly important because they determine their ability to modify flow behavior, impact textural attributes, and stabilize systems under different conditions. Many EPSs exhibit non-Newtonian behavior, such as shear-thinning and thixotropy, making them attractive as structuring agents in food and pharmaceutical formulations. In addition, their thermal properties, including their stability and degradation patterns, further contribute to their suitability for technological applications. The interplay between molecular structure, rheology, and thermal stability underscores the growing relevance of EPSs for applications in food technology, pharmaceuticals, and biomaterials development [4,5,8].

Actinobacteria have attracted increasing interest because of their remarkable capacity to produce structurally diverse and biologically active secondary metabolites. This phylum includes several genera of biotechnological importance, among them Streptomyces, one of the most extensively studied. Actinobacteria have been credited with producing over 30,000 bioactive compounds, encompassing diverse therapeutic classes, including antibiotics, anticancer agents, antifungals, and immunomodulators [5,9]. Complementary estimates from recent reviews indicate that the order Actinomycetales accounts for approximately 45% of all bioactive metabolites discovered. Notably, a substantial proportion of these compounds is produced by a limited number of well-studied genera [9,10,11]. This distribution highlights the need to investigate less-characterized actinobacterial taxa.

Marine environments represent an important reservoir of microbial resources for the discovery of structurally diverse EPS with technological relevance. Marine-derived actinobacteria exhibit adaptive mechanisms to cope with extreme environmental conditions, such as high salinity and nutrient scarcity [12], which can promote the biosynthesis of structurally uncommon EPSs with enhanced thermal stability and biological activity [13]. Many EPSs are heteropolysaccharides that exhibit remarkable structural diversity, with M_w_ ranging from several 10^3^ to over 10^6^ Da [5,14]. Their backbones are composed of diverse monosaccharide residues, including glucose, mannose, xylose, arabinose, rhamnose, fucose, and various uronic and amino sugars [10]. In addition to these common features, marine actinobacteria have been reported to produce EPSs with unusual functional groups and complex branching patterns, which confer distinctive physicochemical properties and broad-spectrum biological activities [9,10]. For example, EPS SRT21, produced by a marine actinobacterial strain isolated from a mangrove environment, demonstrates significant immunomodulatory activity, high thermal stability, and heterogeneous sugar composition [13]. This evidence highlights the biotechnological potential of marine actinobacteria as valuable sources of multifunctional EPS with promising applications in pharmaceutical and biomedical fields.

Despite these advances, the EPS-producing capacity of several rare marine actinobacterial species remains largely unexplored. *Janibacter limosus*, a marine-derived actinobacterium belonging to the family Intrasporangiaceae, was reported by Ettoumi et al. [12] as the dominant culturable actinobacterium in the Tyrrhenian Sea. It exhibits high microdiversity and biosurfactant activity, suggesting substantial adaptive capacity to produce bioactive molecules. However, EPS production by this genus remains poorly documented, and detailed physicochemical characterization of its extracellular polysaccharides is lacking.

In the present study, we report for the first time the isolation and physicochemical characterization of EPS produced by *J. limosus*. To achieve this, JlEPS was extracted, purified, and analyzed using Fourier-transform infrared (FT-IR) spectroscopy, thermogravimetric analysis (TGA), size-exclusion chromatography (SEC) analysis, and monosaccharide composition profiling.

## 2. Materials and Methods

### 2.1. Bacterial Strain, Culture Conditions, and JlEPS Extraction

The *Janibacter limosus* strain used in this study was originally isolated from marine sediments collected from the Tyrrhenian Sea (Italy), as previously described by Ettoumi et al. [12]. The producing strain corresponds to isolate CS 621012, previously identified by partial 16S rRNA gene sequencing and comparison against the GenBank database (BLAST) as reported by Ettoumi et al. [12]; the 16S rRNA gene sequence is available under GenBank accession number KT265291.

The strain was cultivated in mineral medium E*, which contained the following components (g L^−1^): NaCl, 20; (NH_4_)_2_HPO_4_, 3.3; K_2_HPO_4_, 5.8; and KH_2_PO_4_, 3.7. The medium was supplemented with 10 mL of a 100 mM MgSO_4_ solution and 1 mL of a micronutrient solution prepared in 1 N HCl containing (g L^−1^): FeSO_4_·7H_2_O, 2.78; MnCl_2_·4H_2_O, 1.98; CoSO_4_·7H_2_O, 2.81; CaCl_2_·2H_2_O, 1.67; CuCl_2_·2H_2_O, 0.17; and ZnSO_4_·7H_2_O, 0.29 [15]. Glucose was added at an initial concentration of 30 g L^−1^ as the sole carbon source. The inoculum was prepared from 200 mL of a preculture grown in yeast glucose medium containing (g L^−1^): NaCl, 20; MgCl_2_·6H_2_O, 13; MgSO_4_·7H_2_O, 9; KCl, 1.3; CaCl_2_·2H_2_O, 0.2; NaBr, 0.15; NaHCO_3_, 0.05; yeast extract, 0.3; peptone, 0.2; glucose, 30. The cultures were incubated for 48 h at 30 °C and 200 rpm in an orbital shaker, as described by Joulak et al. [16].

Cultivation was performed in fed-batch mode in a 3 L bioreactor (Jupiter 6.0, Solaris Biotechnology Srl, Porto Mantovano, Italy) with a working volume of 2 L. The temperature was maintained at 30 ± 0.2 °C, with the pH controlled at 7.0 ± 0.1 by the automatic addition of 5 M NaOH. Aeration rate was set at 2 standard liters per minute, and dissolved oxygen was maintained at 20% air saturation by automatically adjusting the agitation speed between 200 rpm and 800 rpm. Once this oxygen level was reached, feeding was started by adding 5 mL h^−1^ of a 400 g L^−1^ glucose solution, corresponding to a controlled pulse-feeding strategy. Foam formation was controlled by the automatic addition of antifoam A (Sigma-Aldrich, Taufkirchen, Germany).

During the culture runs, 10 mL samples were collected and centrifuged (12,000× *g* for 30 min, at 4 °C). The biomass pellet was used for the gravimetric determination of cell dry weight (CDW) after washing twice with deionized water (resuspension in water and centrifugation at 11,000× *g* for 15 min). For EPS recovery, the culture supernatant obtained after centrifugation (12,000× *g* for 30 min at 4 °C) was purified by ultrafiltration against distilled water using a Hydrosart ultrafiltration cassette (100 kDa nominal molecular weight cut-off; surface area of 100 cm^2^, Sartorius Stedim Biotech GmbH, Gottingen, Germany), as described by Concórdio-Reis et al. [17]. The concentrated EPS fraction was freeze-dried and used for subsequent analyses.

### 2.2. EPS Characterization

#### 2.2.1. Monosaccharide Composition Analysis

EPS samples (1 mg) were hydrolyzed with 0.1 mL of 99% trifluoroacetic acid (TFA) at 120 °C for 2 h. The hydrolysates were analyzed to identify and quantify the total monosaccharide constituents by HPLC-PAD using a CarboPac PA10 column (Thermo Scientific™ Dionex™, Sunnyvale, CA, USA) equipped with a pulsed amperometric detector, as described by Concórdio-Reis et al. [15]. The analysis was performed at 30 °C with 4 mM NaOH as the eluent, at a flow rate of 0.9 mL min^−1^. Calibration curves were prepared using standard sugars of analytical grade, including glucose (Glc, ≥99%, Sigma-Aldrich, Germany), galactose (Gal, ≥99%, Sigma-Aldrich, Germany), rhamnose (Rha, ≥99%, Fluka Analytical, Buchs, Switzerland), arabinose (Ara, ≥99%, Tokyo Chemical Industry, Tokyo, Japan), galactosamine (GalN, ≥98%, Alfa Aesar, Kandel, Germany), glucosamine (GlcN, ≥99%, Sigma-Aldrich, Germany), fucose (Fuc, ≥99%, Acros Organics, Geel, Belgium), and galacturonic acid (GalA, ≥97%, Fluka Analytical, Buchs, Swizerland). All analyses were performed in duplicate.

#### 2.2.2. Elemental Composition Analysis

JlEPS underwent elemental analysis using an elemental analyzer (Flash EA 1112 CHNS series, Thermo Finnigan-CE Instruments, Rodano, Italy). The nitrogen, carbon, hydrogen, and sulfur content were determined using a thermal conductivity detector after combustion and separation as described by Concórdio-Reis et al. [15].

#### 2.2.3. Molecular Mass Distribution and Polydispersity Determination

The number and average molecular weights (M_n_ and M_w_, respectively) of JlEPS, as well as the polydispersity index (PDI, M_w_/M_n_), were determined using gel permeation chromatography size exclusion chromatography (GPC-SEC), as described by Concórdio-Reis et al. [15]. Analyses were carried out on a KNAUER Smartline HPLC system equipped with a linear LC Phenogel column (300 × 7.8 mm; Phenomenex, Torrance, CA, USA), using 0.1 M LiNO_3_ as the eluent at a flow rate of 0.6 mL min^−1^ at 25 °C. A 50 μL EPS solution (0.5%, *w*/*v*, in 0.1 M LiNO_3_) was injected and detected using a refractive index detector (2414). M_n_ and M_w_ values were calculated from a calibration curve generated using pullulan standards.

#### 2.2.4. Functional Group Analysis by FT-IR Spectroscopy

The functional groups were analyzed by Fourier-transform infrared (FT-IR) spectroscopy, and the spectra were recorded using a Spectrum II spectrometer (Perkin-Elmer, Llantrisant, UK). Spectra were acquired between 500 and 4500 cm^−1^ using 10 scans at room temperature.

#### 2.2.5. Thermal Properties of JlEPS

The thermal properties of the JIEPS samples (10 mg) were investigated using thermogravimetric (TG) analysis using a Labsys EVO thermogravimetric analyzer (Setaram, Caluire, France). The analysis was carried out at a heating rate of 10 °C min^−1^ over a temperature range of 25 to 800 °C under a nitrogen atmosphere.

#### 2.2.6. Rheological Properties of JlEPS

Rheological measurements of JlEPS aqueous solutions at different concentrations (0.5, 1, 3, and 5 wt%) were performed at 25 °C using a controlled-stress rheometer (MCR92 modular compact rheometer, Anton Paar, Graz, Austria) equipped with a Peltier temperature control system and cone-plate geometry (2° angle, 35 mm diameter, and 0.145 mm gap) [18]. The Carreau model (Equation (1)) was applied to describe the flow behavior of JlEPS solutions [18,19].

(1) η=η∞+η0−η∞1+(λγ˙)21−n2,

where η is the apparent viscosity (Pa·s), $\dot{\gamma }$ is the shear rate (s^−1^), *λ* is a time constant (s), *η*_0_ (Pa·s) is the zero-shear rate viscosity (1st Newtonian plateau), *η*_∞_ (Pa·s) is the viscosity of the 2nd Newtonian plateau, and *n* is the viscosity exponent. Since the 2nd Newtonian plateau was never reached, the Carreau equation was simplified by assuming *η*_∞_ ≪ *η*_0_ and *η*_∞_ ≪ η.

## 3. Results and Discussion

### 3.1. JlEPS Production

Glucose served as the carbon substrate for *J. limosus* cultivation during the initial batch phase, primarily supporting biomass formation. This was followed by a fed-batch phase, in which a concentrated glucose solution (400 g L^−1^) was supplied in controlled pulses to sustain metabolic activity and EPS biosynthesis.

*J. limosus* exhibited progressive growth and EPS biosynthesis over a 5-day cultivation period at 30 °C. Biomass accumulation increased progressively, reaching a maximum CDW of 50.7 ± 0.37 g L^−1^ at the end of the cultivation period (117 h). During this period, EPS production peaked at 1.83 ± 0.01 g L^−1^ at 95 h and subsequently reached a plateau during the stationary phase. This corresponds to an average volumetric productivity of approximately 0.019 g L^−1^ h^−1^ during the active production phase.

These results indicate that JlEPS was primarily secreted during the exponential growth phase, which is consistent with the findings reported for other halophilic strains [4,18]. Following this phase, the EPS concentrations stabilized and remained constant throughout the stationary phase. This pattern agrees with previous studies, which highlighted that halophilic bacteria generally achieve maximum EPS yields during active cell growth when glucose serves as the carbon source [4,16]. Compared to previous halophilic EPS-producing strains, *Janibacter limosus* produced JlEPS at levels similar to *Halomonas almeriensis* (1.7 g L^−1^) [19], but six-fold higher than that of *Halomonas ventosae* strains Al12T and Al16 [20], and twenty-four-fold higher than that of *Halomonas smyrnensis* [21]. Although the production level is moderate, it demonstrates that *J. limosus* is capable of synthesizing JlEPS under controlled fed-batch conditions without process optimization, providing a basis for further process improvement.

### 3.2. JlEPS Characterization

#### 3.2.1. Monosaccharide Composition

**Figure 1:**
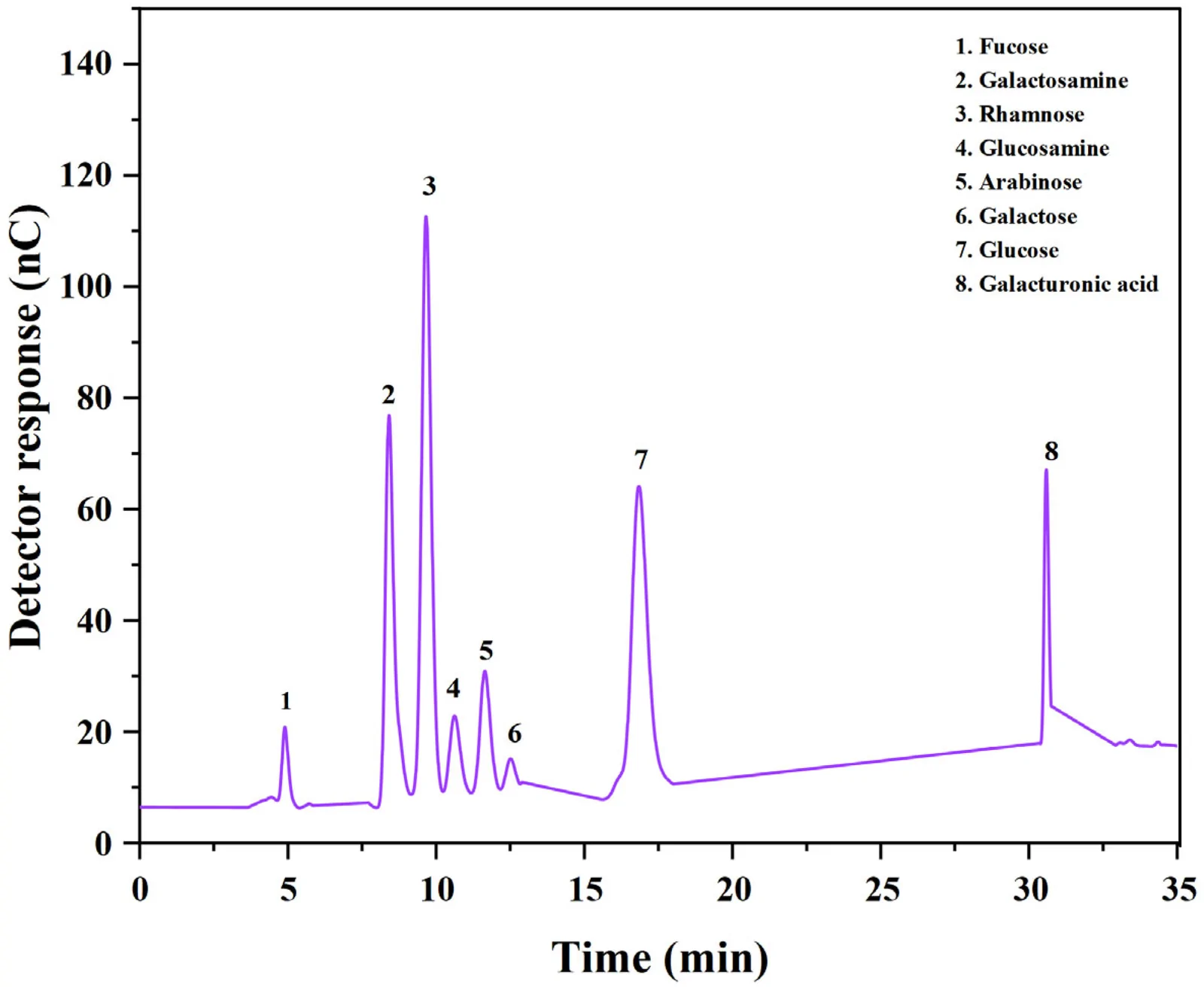
Monosaccharide profile of JlEPS determined by HPAEC-PAD.

Monosaccharide analysis of JlEPS, produced by *Janibacter limosus*, revealed a complex acidic heteropolysaccharide (Figure 1 and Table 1). Quantitative profiling revealed that Glc (36.60 ± 0.46 mol%), Rha (23.62 ± 0.15 mol%), and GalA (16.54 ± 0.18 mol%) were the predominant residues, accounting for over 75% of the total carbohydrate content. GalN (8.70 ± 0.01 mol%) and Ara (7.8 ± 0.76 mol%) were also detected at intermediate levels, whereas GlcN (4.00 ± 0.10 mol%) occurred in lower proportions. Gal (1.35 ± 0.01 mol%) and Fuc (1.42 ± 0.06 mol%) were minor constituents.

The coexistence of neutral, acidic, and amino sugars indicates a structurally heterogeneous polysaccharide architecture with a pronounced polyanionic character arising from the substantial galacturonic acid content. Uronic acid residues introduce ionizable carboxyl groups that can increase charge density along the polymer backbone, potentially promoting electrostatic repulsion, enhanced hydration, and expanded chain conformations in aqueous environments.

In parallel, the presence of amino sugars contributes additional functional diversity that can support hydrogen bonding and charge-mediated intermolecular interactions under suitable physicochemical conditions. Such compositional heterogeneity is a characteristic feature of structurally complex microbial EPS and is frequently associated with distinctive solution behavior and macromolecular organization.

In contrast, exopolysaccharides produced by lactic acid bacteria (LAB), which are among the most extensively characterized microbial polysaccharides, generally display markedly different compositional profiles [22]. Fuso et al. [23] reported that EPS from *L. rhamnosus, L. paracasei*, and *L. bulgaricus* contained glucose and mannose as the dominant sugars, with rhamnose either absent (*L. paracasei*). Amino sugars remained low, while uronic acids were negligible. Against this background, the compositional enrichment observed for JlEPS clearly distinguishes it from the LAB paradigm. Accordingly, JlEPS cannot be readily classified within the structural frameworks commonly described for LAB heteropolysaccharides, which are generally limited to glucose, galactose, and mannose residues, as summarized by Liu et al. [24].

Comparable contrasts are also observed when considering terrestrial bacterial EPS. For instance, the polymer produced by *Streptomyces* sp. SRT21 is predominantly glucose-based with only minor proportions of neutral sugars and uronic acids [13], whereas EPS from *Acidobacteria* strains WH15 and 5B5 described by Kielak et al. [25] consist mainly of glucose, galactose, mannose, and xylose, with trace or undetectable galacturonic acid. These examples collectively illustrate that soil-derived EPS often display simpler compositional patterns, highlighting the comparatively complex and acidic nature of JlEPS.

Interestingly, parallels can be drawn with certain probiotic exopolysaccharides in which compositional attributes have been associated with biological responses. Chen et al. [26] reported that polymers enriched in galactose, rhamnose, and glucosamine enhanced anti-inflammatory responses in macrophage models. Although JlEPS contains only minor amounts of galactose, it combines a high rhamnose fraction with measurable levels of amino sugars, indicating partial compositional similarity to EPS discussed in biological models. However, compositional similarity alone does not demonstrate biological function; therefore, the present work focuses on structure–property relationships. Its additional enrichment in galacturonic acid introduces a substantial density of ionizable carboxyl groups, a structural feature known to increase charge density and promote interactions with cations.

The relationship between acidic composition and extremophilic origin becomes more apparent when considering actinobacteria adapted to marine or halophilic environments. Xiong et al. [27] reported that *Glutamicibacter halophytocola* KLBMP 5180 produced two acidic EPSs enriched in Rha and GalA. EPS-1 consisted of Rha (17.2 mol%), GalA (34.6 mol%), Glc (27.7 mol%), and GlcA (23.5 mol%), whereas EPS-2 contained Rha (11.2 mol%), GalA (35.3 mol%), Glc (13.8 mol%), and Ara (23.2 mol%). Such compositional profiles support the broader observation that actinobacteria inhabiting saline environments frequently synthesize structurally complex acidic polymers. JlEPS shares these compositional characteristics but differs in the presence of amino sugars, particularly galactosamine, which were not reported for the *Glutamicibacter* polymers. The coexistence of uronic acids and amino sugars increases functional group diversity and may influence intermolecular interactions, hydration behavior, and associations with charged molecules in solution.

This alignment is consistent with the trends highlighted by Delbarre-Ladrat et al. [28] and Wei et al. [5], who identified uronic acids, sulfate groups, and heterogeneous substitutions as recurrent features of marine EPS and often discussed them in relation to antioxidant, emulsifying, and immunomodulatory activities. With substantial uronic acid content and a significant amino sugar fraction, JlEPS appears compositionally closer to marine or halophilic EPS than to neutral LAB or simpler soil-derived biopolymers. Its compositional profile integrates several uncommon traits, namely glucose–rhamnose predominance, marked uronic acid enrichment, and measurable amino sugar content, traits seldom observed together within a single bacterial EPS. Collectively, these characteristics indicate that JlEPS occupies a distinct compositional niche among microbial polysaccharides and highlight its relevance as a structurally atypical EPS. Further studies will be necessary to determine how these compositional features translate into specific physicochemical properties.

#### 3.2.2. Elemental Composition

Elemental analysis of JlEPS (Table 1) yielded C, H, N, and S contents of 33.91 ± 1.12 wt%, 5.56 ± 0.28 wt%, 5.39 ± 0.87 wt%, and 0.32 ± 0.20 wt%, respectively. The carbon and hydrogen fractions are consistent with a carbohydrate-dominated matrix enriched in hydroxylated functionalities. The low sulfur content indicates that sulfate ester groups are negligible, supporting the conclusion that the anionic character of JlEPS arises predominantly from uronic acid residues.

The nitrogen content is markedly higher than typically reported for extensively purified bacterial exopolysaccharides (<1–2 wt%) [29]. Monosaccharide analysis showed that amino sugars (galactosamine and glucosamine) represent 12.7 mol% of total residues. Considering that glucosamine contains one nitrogen atom per monomer (≈7.8 wt% N), the theoretical nitrogen contribution from backbone aminosugar incorporation corresponds to approximately 1 wt%. The experimentally determined nitrogen content (5.39 ± 0.87 wt%), therefore, substantially exceeds the fraction attributable to amino sugars alone [30].

Because elemental analysis provides a bulk compositional measurement that does not distinguish between covalently bound nitrogen and associated nitrogen-containing species [31], the elevated nitrogen level is most consistent with contributions from both structural aminosugar residues and co-retained nitrogenous macromolecules present in the recovered extracellular fraction. Such coexistence of polysaccharidic and proteinaceous components is consistent with the structural organization of extracellular polymeric matrices, which are known to form composite assemblies rather than chemically uniform polymers [32]. Further purification and fractionation analyses will be necessary to resolve the relative contributions of these components and will be addressed in future work.

#### 3.2.3. Molecular Mass Distribution

**Figure 2:**
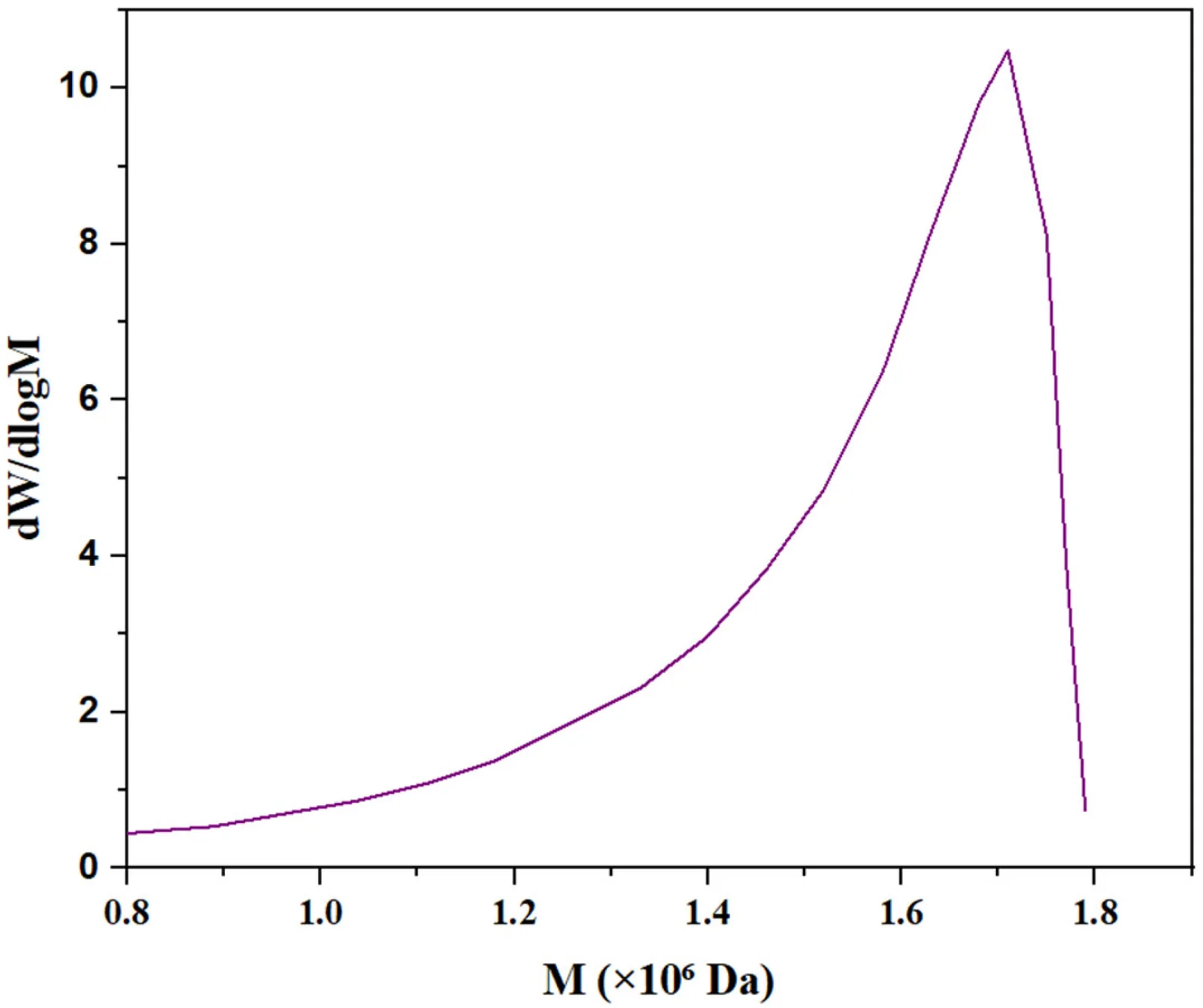
M_w_ distribution curve of JlEPS.

Understanding the molecular weight distribution of exopolysaccharides is essential because polymer chain length and dispersity directly influence macromolecular conformation, intermolecular interactions, and functional performance (Figure 2 and Table 1) [8]. The weight-average (M_w_) of JlEPS was 1.60 × 10^6^ ± 0.04 Da, while the number-average molecular weight (M_n_) was 1.57 × 10^6^ ± 0.06 Da, resulting in a polydispersity index (PDI) of 1.02 ± 0.01. This narrow dispersity reflects a highly uniform macromolecular population with limited chain-length heterogeneity. As previously reported, the EPS corresponds to a polysaccharide–protein-associated complex; therefore, the measured M_w_ represents the overall assembly rather than the carbohydrate backbone alone. A molecular weight in the megadalton range is expected to increase hydrodynamic volume and favor intermolecular entanglement and transient network formation in aqueous systems, parameters that are directly relevant to the rheological performance discussed in subsequent sections. Although most actinobacterial EPS typically fall within the 10^3^–10^5^ Da range, isolated megadalton-scale examples have been reported [5]; thus, JlEPS lies at the upper boundary of the described spectrum.

As summarized by Wei et al. [5], most actinobacterial exopolysaccharides exhibit M_w_ values within the 10^3^–10^5^ Da range, including EPSST from *Streptomyces* MSAHM1 (9.7 × 10^3^ Da) and EPS-1/EPS-2 from *Glutamicibacter halophytocola* KLBMP 5180 (10^5^ Da range). Megadalton-scale polymers have been reported less frequently, such as HPS from *Rhodococcus erythropolis* HX-2 (1.04 × 10^6^ Da) and EPS FR2 from *R. erythropolis* PR4 (~2.00 × 10^6^ Da) [33]. Additional examples, including EPS from *Streptomyces vinaceusdrappus* AMG31 (5.1 × 10^4^ Da) [34], *Streptomyces* sp. SRT21 (2.86 × 10^5^ Da) [13], and *Kocuria* sp. clone Asker4 (1.18 × 10^5^ Da) [35], confirm that moderate M_w_ values predominate within this phylum. Among halophilic actinobacteria, *Glutamicibacter halophytocola* KLBMP 5180 produces EPS of 58.9 kDa [27], indicating that extreme salinity alone does not inherently promote extensive chain elongation. Marine halophiles outside Actinobacteria also predominantly synthesize moderate-M_w_ polymers, including *Virgibacillus dokdonensis* VITP14 (M_w_ 555.05 kDa; M_n_ 479.70 kDa; Đ 1.16) [36], *Vreelandella titanicea* Zn11_249 (3.9 × 10^4^–5.89 × 10^4^ Da) [37], *Alteromonas* sp. JL2810 (1.67 × 10^4^ Da), Bacillus sp. H5 (8.9 × 10^4^ Da), and *Halobacillus* sp. EG1HP4QL (1.10–2.29 × 10^5^ Da) [38,39,40]. Within this comparative framework, JlEPS lies at the upper boundary of the reported spectrum but remains within the range documented for high-molecular-weight representatives.

The elevated M_w_ of JlEPS, approximately one order of magnitude higher than the typical actinobacterial range, likely reflects strain-specific regulation of polymer chain propagation and termination. Its compositional profile, characterized by substantial neutral sugars together with uronic and amino sugar residues, supports a structurally heterogeneous yet chemically coordinated backbone. Uronic acids contribute ionizable carboxyl groups, while amino sugars introduce nitrogen-containing functionalities, enabling electrostatic interactions and hydrogen bonding along extended chains. In a high-M_w_ system with restricted dispersity, such functional group distribution is consistent with regulated chain elongation, limited premature termination, and stabilization of intermolecular associations. These features are consistent with the possibility of regulated glycosyltransferase activity and controlled precursor flux, which together could underpin both polymerization efficiency and architectural uniformity.

From a functional standpoint, high-molecular-weight EPS are frequently associated with enhanced rheological performance, viscoelasticity, hydration capacity, and film-forming behavior [8,41,42,43,44]. Such physicochemical attributes have also been correlated with biological activities, including radical scavenging and immune modulation [5], although these relationships depend on specific structural determinants and require targeted mechanistic validation. Collectively, the combination of high M_w_, low dispersity, and chemically functionalized backbone delineates JlEPS as a structurally differentiated exopolysaccharide within the actinobacterial repertoire and provides a coherent structural basis for its observed physicochemical properties.

#### 3.2.4. Functional Group Analysis

**Figure 3:**
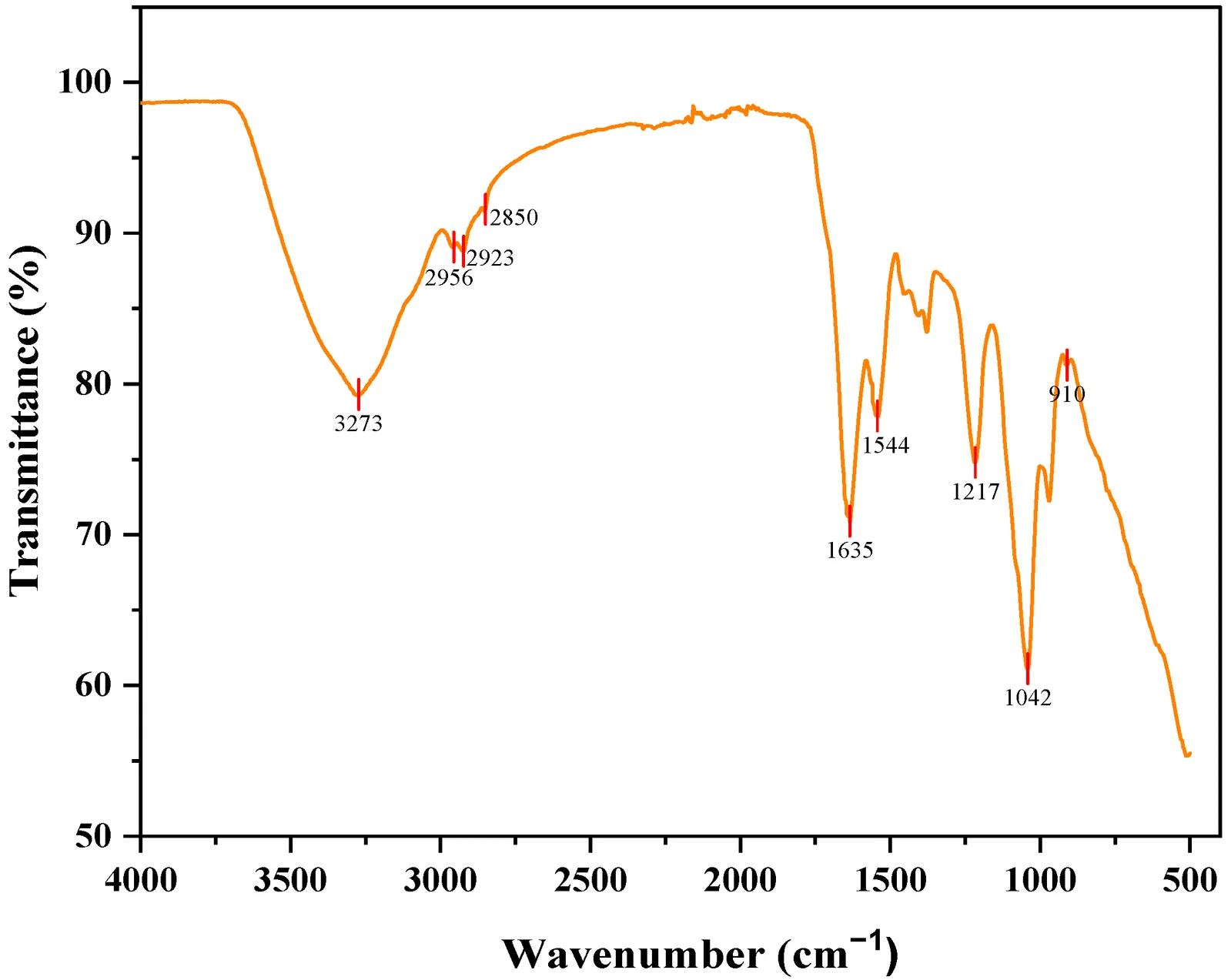
FT-IR spectrum of JlEPS produced by the strain *Janibacter limosus*.

The functional group profile of JlEPS was determined using FT-IR analysis, which revealed characteristic absorption bands typical of polysaccharides. Figure 3 shows that the spectrum exhibited signals consistent with those commonly reported for carbohydrate-based polymers. The broad, intense band observed at 3273 cm^−1^ corresponds to O–H stretching vibrations of hydroxyl groups, indicating the presence of a polysaccharide backbone and extensive hydrogen bonding. This observation is consistent with the high abundance of hydroxylated monosaccharides, such as glucose and rhamnose, identified in the compositional analysis. The C–H stretching bands at approximately 2956, 2923, and 2850 cm^−1^ [15,45] are attributed to aliphatic CH_2_ groups and are commonly detected in halophilic EPSs, including those produced by *Vreelandella titanicae* Zn11_249 [37] and *Chromohalobacter salexigens* strain 3EQS1 [46], suggesting comparable structural features within the polymer backbone.

The absorption peak at 1635 cm^−1^ corresponds to C=O stretching vibrations, consistent with the presence of carboxyl groups and supporting the detection of uronic acid residues in the monosaccharide profile [47]. Such ionizable functionalities are known to promote intermolecular electrostatic interactions and hydration, providing a plausible structural basis for polymer cohesion and the elevated molecular weight observed for JlEPS. The weak band at 1544 cm^−1^ is assigned to N–H deformation vibrations, indicating the presence of nitrogen-containing functionalities within the recovered extracellular fraction, consistent with the detection of amino sugars in the compositional analysis.

The bands at 1452, 1406, and 1377 cm^−1^ correspond to C–H bending vibrations of CH_2_ and CH_3_ groups, which are typical features of polysaccharide structures. The absorption at 1217 cm^−1^ is attributable to overlapping contributions from S=O stretching and/or C–O–C vibrations [45]; considering the negligible sulfur content determined by elemental analysis, this region is more plausibly associated with glycosidic or ether linkages rather than sulfated substituents. The strong peak at 1042 cm^−1^ was assigned to C–O and C–C vibrations within glycosidic bonds and pyranose rings [6,15], supporting the integrity of the carbohydrate backbone and the heteropolysaccharide nature of JlEPS. The weak band at 910 cm^−1^ was attributed to β-configuration vibrations of the glucopyranose ring, consistent with the presence of β-glycosidic linkages in the polymer structure. Similar absorptions associated with β-linkages have been reported for other structurally stable bacterial heteropolysaccharides [42].

Overall, the FT-IR spectrum is consistent with the compositional and molecular-weight data, indicating that JlEPS is a hydroxyl-rich, carboxyl-containing heteropolysaccharide capable of extensive hydrogen bonding and intermolecular association. This functional group distribution provides a coherent structural basis for the macromolecular organization and physicochemical behavior expected for high-molecular-weight microbial EPS.

#### 3.2.5. Thermal Properties

**Figure 4:**
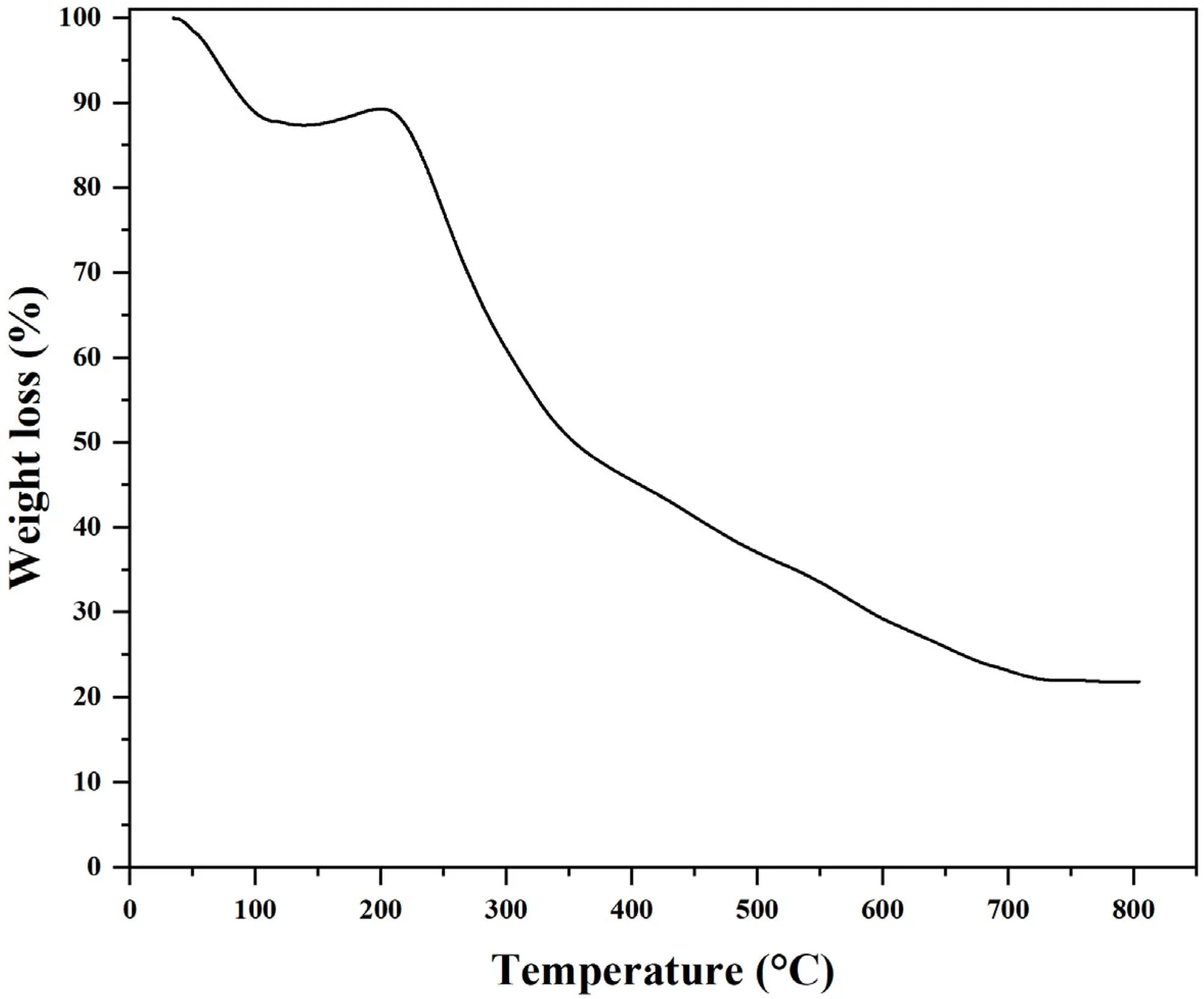
Thermogravimetric analysis of JlEPS.

The thermal behavior of JlEPS was evaluated by thermogravimetric analysis (TGA) (Figure 4), revealing a two-step degradation profile typical of structurally organized polysaccharides. In the first stage (35–140 °C), a 13% mass loss was observed and attributed to the release of physically bound water. This dehydration step is consistent with a polymer matrix rich in hydrophilic functional groups capable of hydrogen bonding, such as hydroxyl and carboxyl moieties, in agreement with the monosaccharide composition and FT-IR results showing abundant hydroxylated sugars and uronic acid residues that enhance water-retention capacity [8,48,49,50].

A second major weight loss of 67.3% occurred between 200 and 757 °C, corresponding to decomposition of the polysaccharide backbone and side chains. This stage reflects cleavage of glycosidic linkages and fragmentation of the polymer network and represents the upper thermal stability limit of JlEPS. The material remained stable up to approximately 250 °C, indicating notable thermal resistance for a microbial polysaccharide. Such stability is consistent with the structural features identified in previous analyses, including high molecular weight, narrow dispersity, and the presence of uronic acid residues, all of which are known to promote intermolecular interactions and cohesive polymer architectures that delay thermal degradation [49,50].

SRT21-EPS exhibited earlier thermal transitions at 138 °C and 164 °C, followed by major decomposition events at 294 °C and 301 °C, with a final residue of 52.2% at 800 °C [13]. These data indicate that although both polymers exhibit strong thermal resistance, JlEPS maintains structural integrity over a broader temperature range. Similarly, the EPS reported by Mosawi et al. [51] showed a two-step degradation pattern with major weight loss near 280 °C, consistent with the behavior observed here. Such similarities suggest that marine bacterial EPS commonly exhibit comparable degradation profiles due to their heterogeneous architectures and stabilizing functional groups.

The thermal profile of JlEPS agrees with those reported for EPS produced by *Alteromonas* sp. [8], *Halomonas* strains (*H. caseinilytica* K1, *H. elongata* K4, *H. smyrnensis* S3, *H. halophila* S4) [52], and *Streptomyces griseorubens* GD5 [53], indicating that its thermal response falls within the expected stability range of structurally complex microbial polysaccharides.

#### 3.2.6. Rheological Properties

**Figure 5:**
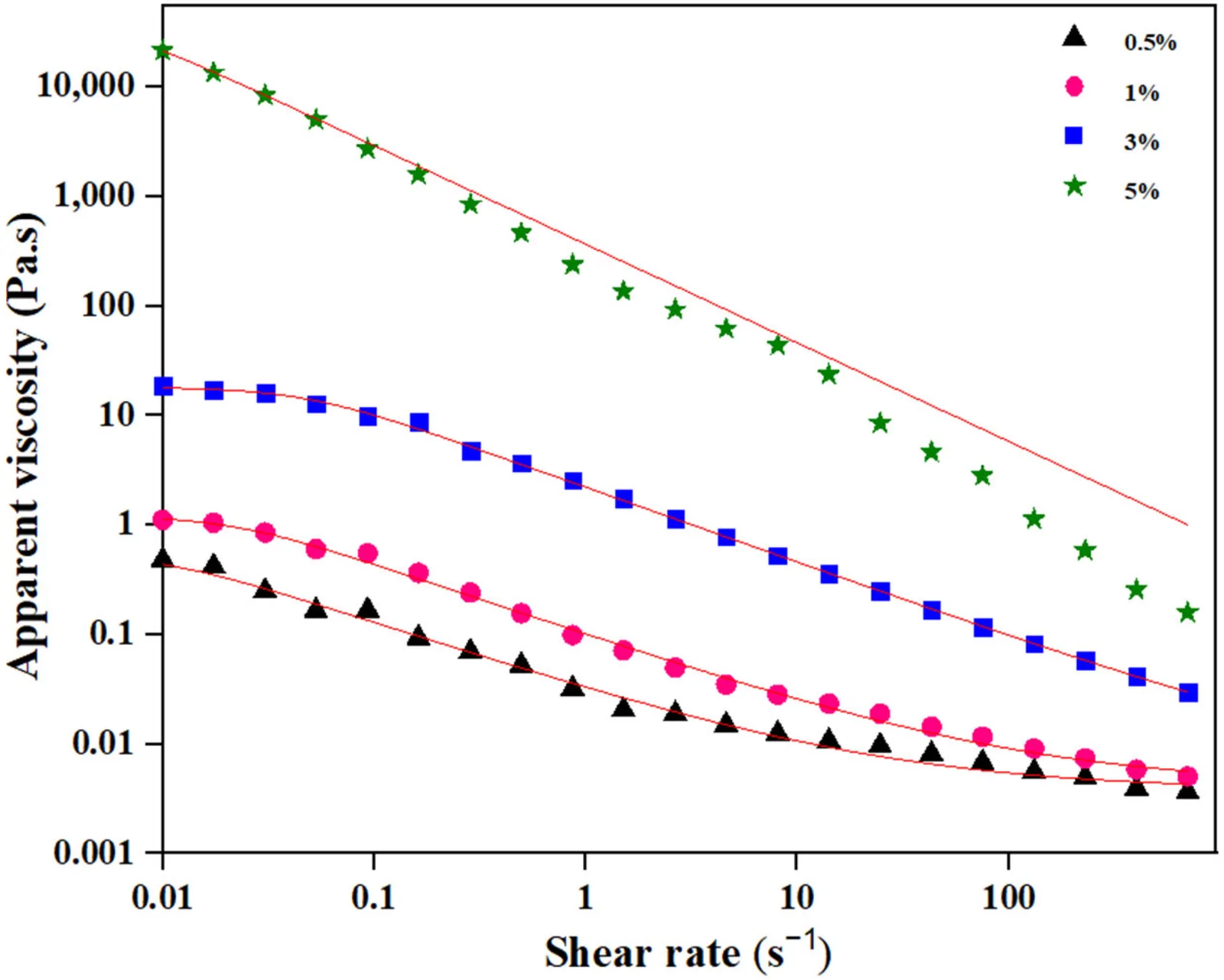
Apparent viscosity as a function of shear rate of JlEPS at different concentrations (0.5–5 wt%) fitted with the Carreau model.

The flow curves in Figure 5 show the apparent viscosity of JlEPS solutions (0.5–5 wt%) as a function of shear rate. Increasing polymer concentration led to a strong rise in viscosity at constant shear (0.1 s^−1^), from 0.542 ± 0.187 Pa·s to (5.01 ± 0.91) × 10^4^ Pa·s, indicating pronounced concentration-dependent thickening. At all concentrations, viscosity decreased as shear rate increased, confirming shear-thinning behavior. This response is typical of structured polymer solutions in which intermolecular interactions—such as hydrogen bonding, electrostatic forces, and chain entanglements—form transient networks that resist flow at low shear but progressively align and disentangle under higher shear, reducing viscosity [54,55]. Similar behavior has been reported for other microbial EPS systems [8,54,56,57], including polymers from *Alteromonas* strains [8], soluble polysaccharides from *Chorella vulgaris* [48], and EPS produced by *Rhizobium viscosum* CECT908 [58].

The correlation between the apparent viscosity and shear rate was further described using the Carreau model [59]. As shown in Table 2, a Newtonian plateau at low shear rates was followed by pronounced shear-thinning behavior observed on the various concentration curves at high shear rates. The results showed that increasing the JlEPS concentration from 0.5 to 5 wt% led to an increase in *η*_0_ from 0.542 ± 0.187 Pa·s to (5.01 ± 0.91) × 10^4^ Pa·s, respectively, and was accompanied by a progressive decrease in the viscosity exponent (*n*) from 0.368 ± 0.032 to 0.100 ± 0.028. These changes reflect enhanced molecular interactions and the formation of entangled networks in high-concentration solutions, resulting in more interactions and entangled aggregates, thereby increasing flow resistance and viscosity [54,55,60]. The high R^2^ values (0.965–0.999) suggest that the Carreau model adequately represents the experimental data, confirming its suitability for describing the rheological behavior of JlEPS solutions.

Comparison with other microbial EPS highlights the strong thickening efficiency of JlEPS. Concórdio-Reis et al. [54] reported that *η*_0_ of EPS from *Altermonas macleodii* Mo169 increased from 1.38 ± 0.02 Pa·s at 0.18 wt% to 156 ± 4 Pa·s at 1.25 wt%, highlighting the strong dependence of viscosity on polymer concentration. Similarly, Xu et al. [61] observed that the *η*_0_ of EPS-3 from *Streptococcus thermophilus* S-3 increased from 0.14 Pa·s at 2 wt% to 2.5 Pa·s at 4 wt%. Notably, the viscosity of JlEPS is markedly higher than values reported for EPS22 from *Pseudomonas stutzeri* AS22, which reached up to 102 Pa·s at a concentration ≤ 1 wt% [41], or for EPS from *Lactobacillus rhamnosus* E/N, which showed a viscosity of 5–25 mPa·s at 1 wt% [62]. This rheological behavior is consistent with structural features established in preceding sections. The combination of high molecular weight, low dispersity, and enrichment in uronic acid and rhamnose residues provides a plausible structural basis for enhanced intermolecular association and chain expansion in solution. Such attributes are widely recognized to promote entanglement, hydration, and network formation, thereby explaining the strong viscosity increase and pronounced shear responsiveness observed for JlEPS.

## 4. Conclusions

This study reports the characterization of a previously undescribed exopolysaccharide produced by *Janibacter limosus* and defines its principal compositional and physicochemical features. Monosaccharide profiling revealed a heterogeneous acidic polymer dominated by glucose, rhamnose, and galacturonic acid, indicating a structurally complex heteropolysaccharide. FT-IR spectroscopy confirmed functional groups consistent with a polysaccharide backbone, including hydroxyl, carboxyl, and glycosidic moieties. M_w_ analysis showed a high M_w_ (~1.6 × 10^6^ Da) with low dispersity, indicating a relatively uniform macromolecular population. Thermal analysis demonstrated stability up to approximately 250 °C, and rheological measurements revealed strong concentration-dependent viscosity together with pronounced shear-thinning behavior.

Taken together, these results establish JlEPS as a structurally distinctive, high-molecular-weight polymer whose compositional profile and macromolecular properties differ from those of most previously described actinobacterial exopolysaccharides. The experimentally determined nitrogen content exceeded the theoretical value expected for a purified polysaccharide backbone, suggesting the presence of associated nitrogen-containing components within the recovered extracellular fraction rather than solely intrinsic structural substitution. Such compositional complexity is consistent with the known organization of extracellular polymeric matrices and does not affect the structural assignments derived from chromatographic and spectroscopic analyses.

Overall, this work introduces JlEPS as a novel actinobacterial exopolysaccharide and provides a physicochemical framework for its classification. The results establish a foundation for future targeted structural elucidation and expanded structure–function relationships.

## Figures and Tables

**Table 1:** Molecular distribution, monosaccharide composition, and elemental analysis of JlEPS.

Molecular Distribution	
M_w_	1.60 × 10^6^ ± 0.04 Da
M_n_	1.57 × 10^6^ ± 0.06 Da
PDI	1.02 ± 0.01
Monosaccharide Composition	
Glucose	36.60 ± 0.46 mol%
Rhamnose	23.62 ± 0.15 mol%
Galacturonic acid	16.54 ± 0.18 mol%
Galactosamine	8.70 ± 0.01 mol%
Arabinose	7.8 ± 0.76 mol%
Glucosamine	4.00 ± 0.10 mol%
Galactose	1.35 ± 0.01 mol%
Fucose	1.42 ± 0.06 mol%
Elemental Analysis (CHNS)	
Carbon (C)	33.91 ± 1.12 wt%
Hydrogen (H)	5.56 ± 0.28 wt%
Nitrogen (N)	5.39 ± 0.87 wt%
Sulfur (S)	0.32 ± 0.20 wt%

**Table 2:** Carreau model parameters estimated for JlEPS solutions at different concentrations (0.5–5 wt%) produced by the strain *J. limosus*. *η*_0_: zero-shear viscosity; *λ*: relaxation time; *n*: flow behavior index; R^2^: coefficient of determination.

Concentration (wt%)	*η*_0_ (Pa·s)	*λ* (s)	*n*	R^2^
0.5	0.542 ± 0.187	100 ± 68.0	0.368 ± 0.032	0.965
1	1.213 ± 0.136	47.41 ± 12.22	0.346 ± 0.022	0.984
3	1.806 ± 0.627	21.30 ± 1.76	0.313 ± 0.008	0.997
5	(5.01 ± 0.91) × 10^4^	236.4 ± 26.61	0.100 ± 0.028	0.999

## Data Availability

The original contributions presented in this study are included in the article. Further inquiries can be directed to the corresponding author.
